# Willingness to work during initial lockdown due to COVID-19 pandemic: Study based on an online survey among physicians of Bangladesh

**DOI:** 10.1371/journal.pone.0245885

**Published:** 2021-02-09

**Authors:** Md. Abdur Rafi, M. Tasdik Hasan, Dewan Tasnia Azad, Syeda Fatema Alam, Vivek Podder, Sahadat Hossain, S. M. Quamrul Akther, Fatema Ashraf, Md. Golam Hossain

**Affiliations:** 1 Rajshahi Medical College, Rajshahi, Bangladesh; 2 Department of Primary Care and Mental Health, University of Liverpool, Liverpool, United Kingdom; 3 Public Health Foundation, Dhaka, Bangladesh; 4 Jashore Medical College, Jashore, Bangladesh; 5 Shaheed Suhrawardy Medical College Hospital, Dhaka, Bangladesh; 6 Tairunnessa Memorial Medical College and Hospital, Gazipur, Bangladesh; 7 Department of Public Health and Informatics, Jahangirnagar University, Dhaka, Bangladesh; 8 Department of Statistics, Health Research Group, University of Rajshahi, Rajshahi, Bangladesh; Qazvin University of Medical Sciences, ISLAMIC REPUBLIC OF IRAN

## Abstract

**Background:**

During the catastrophic situation of the COVID-19 pandemic, the role of the health care workers (HCWs) is the most crucial, and their absenteeism, whether due to inability or unwillingness, becomes a major concern for the national health system. Hence, the present study aimed to determine the willingness and its associated factors to work during the COVID-19 pandemic among the physicians of Bangladesh.

**Methods:**

This was a cross-sectional study conducted from April 21 to May 10, 2020, using an online survey among the Bangladeshi physicians living in the country. Both univariate and multivariable binary logistic regression models were used to determine the predictors of the willingness of the physicians to work during the COVID-19 pandemic.

**Results:**

More than 69% physicians reported that they were willing to work during the COVID-19 pandemic, 8.9% reported that they were not willing, while 21.4% of participants were not sure about their willingness. Younger age, having experience of treating patients during previous pandemics, working in the emergency departments and high self-reported compliance to the recommended PPE were important predictors of being willing to work during COVID-19 pandemic. Concern for family and risk of transmitting the infection to family members were most commonly reported as major barriers of working during the pandemic (30%) followed by having comorbidities (25%), lack of adequate safety measures (25%), fear of being infected (12.2%), not involved in clinical practice (12.5%) etc.

**Conclusions:**

Though the majority of the physicians were willing to work during the COVID-19 pandemic, sufficient supply of PPE, support to maintain recommended quarantine and isolation policy after risky hospital duty along with adequate and effective training can increase their willingness to continue their sacred duty during this crucial pandemic.

## Background

Since its emergence in early 2020, the COVID-19, a highly contagious respiratory infection caused by severe acute respiratory syndrome coronavirus 2 (SARS-CoV-2), has become a global health threat [1, 2]. More than ten million people have been affected by COVID-19 resulting in more than half a million death worldwide by the last week of July 2020 and the number has been increasing exponentially [3]. The resilience of the health care systems of many affected countries has been threatened and close to collapse due to the large number of cases requiring both outpatient and intensive care services [4–6]. The threats are more extensive for the fragile health care systems of low and middle-income countries like Bangladesh where the healthcare system remains brittle to cope with the sudden rush of cases during a pandemic [7–9].

Health care workers (HCWs) are the frontline professionals to respond during the crucial situation of the pandemic. While millions of people are staying at home globally to minimize the spread of SARS-CoV-2 infection, the HCWs are putting themselves at high risk of getting infected in hospitals. In addition to the risk of infection, the physical and mental exhaustion, distress of complex triage decisions, and the grief of losing patient and colleagues, poor access to personal protective equipment (PPE), and anxiety of passing the infection to their family have confronted them with greater uncertainty [10]. More than 90,000 HCWs worldwide have been infected with COVID-19, and possibly twice that are in shortages of protective equipment [11]. As a result, not all HCWs will be able to continue their work during the pandemic due to a range of factors including being infected or for their potential high health risk due to pre-existing comorbidities or due to performing as caregivers for vulnerable or infected family members [12, 13]. Moreover, some HCWs might not be willing to continue work amid such a crisis, even if being physically capable to do so. Though the righteousness of this reluctance of the HCWs is debatable, it is assumed that their absenteeism would increase during the pandemic situation [12, 14]. For instance, the potential levels of absenteeism during an epidemic has been reported as 16% in Hong Kong [15], 28% in Germany [16], 33% in Australia [17], 43% in Taiwan [18] and 50% in the UK [19]. A number of factors are associated with this reluctance to work of the HCWs including high perceived self or family members’ risk of being infected, personal health issues, lack of proper personal protective equipment and facilities in the workplace as well as lack of knowledge and confidence about the pandemic [13, 20, 21].

The inability or reluctance of the HCWs to continue work during a pandemic is a major challenge to keep the health system functioning, especially for limited resources countries like Bangladesh, which are already running short of an adequate number of health care providers. So, it is important to determine the prevalence and barriers of willingness to work of the HCWs during a pandemic for taking further actionable plans to remove the barriers. Therefore, this study aimed to find out the prevalence and associated factors of willingness to work during the COVID-19 pandemic among the registered physicians of Bangladesh, the major part of the healthcare workers of the country.

### Research questions

There are two main research questions of this study such as (i) how many physicians in Bangladesh are not interested to do willing work at their working place during initial lockdown due to COVID-19 pandemic? (ii) what are the associated factors of compliance to work during the COVID-19 pandemic?

## Methods

### Ethics statement

The research protocol was reviewed and approved (ShSMCH/Ethical/2020/12) by the Ethical Review Committee, Shaheed Suhrawardy Medical College, Dhaka, Bangladesh. We got e-mail ID of the participants from their completed questionnaire, and took their written consent through e-mail.

### Study design

This was a cross-sectional type of observational study, conducted from April 21 to May 10, 2020, when the COVID-19 pandemic and the consequential lockdown was in its initial phase in the country with an increasing number of cases from three thousand to fifteen thousand during the study period [3].

### Sample size determination

All the registered physicians in Bangladesh were the study population. The sample size was calculated from the prevalence estimate using the formula: $n=\frac{z^{2}p(1-p)}{d^{2}}$, where, where n = number of the sample; z = 1.96 for 95% confidence interval (CI), p = “best guess” for prevalence and d = precision of the prevalence estimate. There is no existing data on willingness to work during a pandemic among the physicians of Bangladesh. However, a review reported that the rate varies from 23% to 96% depending on context [21]. We assumed that the rate of willingness to work during a pandemic would be 50% among the physicians of Bangladesh and it provided that 384 samples would be enough for the present study. Assuming a 10% non-response rate, initially, we considered the sample size as 422.

### Sampling and data collection procedure

We collected data from physician during COVID-19 pandemic, could not possible to directly contact them considering the risks associated with face to face data collection approach, data were collected through online. An online survey was posted on closed social media (Facebook) groups of registered Bangladeshi physicians living in this country. Five volunteers from different medical institutions were employed to circulate the survey among their professional networks in addition to regular posting in the above-mentioned social media groups. They were instructed to be inclusive, open, and circulate it periodically for maximum reach. We did not have any ID list of all registered Bangladeshi physicians living in this country; probability sampling could not possible to apply for this survey. We used both convenient and snowball sampling methods to recruit participants, where physicians known by the volunteers were first contacted, and an open request was placed by the team of investigators to fill-up the form. The physicians were recruited through an electronic questionnaire on Google Drive ®. Once the questionnaire was completed, they were asked to circulate other physicians of their contacts and so on, until completed our required sample 422, but unfortunately 109 physicians did not post their completed form. Finally, 313 physicians were considered as sample for the present study. Later the email addresses of the participants were collected from their completed questionnaire, and took their consent for the publication of the data. The study was conducted following the Checklist for Reporting Results of Internet E-Surveys (CHERRIES) guidelines [22].

### Data collection instrument

Data were collected using a pre-tested, structured online questionnaire created in Google form. The questionnaire had three parts: (i) socio-demographic and professional information of the participants, (ii) knowledge, attitude and practice of using the WHO recommended PPE against SARS-CoV-2, and (iii) willingness to work during the pandemic.

#### Part 1: Socio-demographic information

This part included the questions about the socio-demographic (such as age, sex, marital status, etc.), profession, and workplace-related characteristics (such as professional experience, training, and experience of handling COVID-19 patients, facilities of the working hospital, etc.) of the participants.

#### Part 2: Knowledge, attitude, and practice of using the PPE

The knowledge about PPE was assessed by a 13-item questionnaire on correct components of PPE (7 items), hand hygiene (3 items), right PPE during providing direct care, performing the aerosol-generating procedure and consulting patients with respiratory symptoms in outpatients setting (3 items) based on the WHO guideline [23]. The total possible score was 13. Those who scored above 80% (≥11 out of 13) were considered as having adequate knowledge.

The attitude towards PPE was assessed by a 5-item questionnaire tailored based on previous studies [24, 25]. The confidence of understanding the risk and protective measures of COVID-19 for health care professionals as well as patients, perceived protection from COVID-19 of HCWs and patients by using the PPE and convenience of using those was assessed. A five-point Likert scale from ‘completely agree’ to ‘completely disagree’ was used for evaluation of the attitude. Those who selected ‘completely agree’ or ‘agree’ for the attitude statements were considered as ‘agree’, while others were considered as disagree [25]. An additional assessment of the self-perceived risk of being infected by SARS-CoV-2 was measured using a single item question, ‘How much do you feel to be affected by COVID-19 during your hospital work?’ The self-reported risk of being affected ≥6 on a linear scale of 10 was considered as a high perceived risk.

The practice of using PPE was assessed based on the WHO guideline. Using medical mask, gown, gloves, and eye protection during providing direct care to COVID-19 patients or consulting a patient with respiratory symptoms at outpatient, Respirator N95 along with mentioned others during performing aerosol-generating procedures on COVID-19 patients was considered as an adequate practice of using PPE. For surgical and gynecological procedures on normal patients without respiratory symptoms, surgical musk was considered as safe. Cloth masks or other masks were not considered as PPE [23]. Self-reported compliance to PPE use of ≥8 on a linear scale of 10 was considered as high compliance based on the evidence of previous studies [24, 25].

#### Part 3: Willingness to work

Willingness to work during the pandemic was assessed using a single item question, ‘Are you willing to work in your hospital during the COVID-19 pandemic?’ Those who responded ‘Yes’ were considered as willing to work and those who responded ‘No’ or ‘Not sure’, were considered as not willing.

### Outcome variable

Willingness to work during initial lockdown due to the COVID-19 pandemic was the outcome variable of the study. It was classified into two categories such as (i) yes (code, 1) and (ii) no (code, 0). Response as ‘No’ and ‘Not sure’ both were included in the ‘no’ category.

### Independent variables

Respondents’ socio-demographic and profession related characteristics, knowledge, attitude, and practice of using protective equipment and perceived risk of being infected by SARS-CoV-2 were the independent variables. All independent variables with their categories are mentioned in Table 1. Knowledge, attitude, and practice related variables are mentioned in Table 2.

**Table 1:** Socio-demographic and professional characteristics of the participants (n = 313).

Characteristics	Total	Willing,	Not willing,	Uncertain,	p-value
		n (%)	n (%)		
				n (%)	
*Socio-demographic characteristics*					
Age (years) (Mean = 30.97, SD = 7.0)					
21–30	215	155 (72.1)	17 (7.9)	43 (20.0)	0.070^a^
31–40	57	42 (73.7)	6 (10.5)	9 (15.8)	
>40	41	21 (51.2)	5 (12.2)	15 (36.6)	
Sex					
Male	142	109 (76.8)	15 (10.6)	18 (12.7)	0.003
Female	171	109 (63.7)	13 (7.6)	49 (28.7)	
Marital status					
Married	188	128 (68.1)	24 (12.8)	36 (19.1)	0.011^a^
Unmarried	125	90 (72.0)	4 (3.2)	31 (24.8)	
Cohabitation					
With parents	120	90 (75.0)	5 (4.2)	25 (20.8)	0.026^a^
With spouse only	58	43 (74.1)	6 (10.3)	9 (15.5)	
With spouse and children	85	54 (63.5**)**	14 (16.5)	17 (20.0)	
Alone	50	31 (62.0)	3 (6.0)	16 (32.0)	
*Profession related characteristics*					
Professional status					
Consultant	50	23 (46.0)	8 (16.0)	19 (38.0)	0.002^a^
Early career physician	230	171 (74.3)	19 (8.3)	40 (17.4)	
Intern physician	33	24 (72.7)	1 (3.0)	8 (24.2)	
Professional qualification					
Graduate	227	165 (72.7)	19 (8.4)	43 (18.9)	0.154
Postgraduate	86	53 (61.6)	9 (10.5)	24 (27.9)	
Professional experience					
Up to 1 year	62	41 (66.1)	4 (6.5)	17 (27.4)	0.007^a^
2–5 years	159	124 (78.0)	10 (6.3)	25 (15.7)	
More than 5 years	92	53 (57.6)	14 (15.2)	25 (27.2)	
Experience of treating confirmed or suspected COVID-19 patients					
Yes	64	37 (57.8)	7 (10.9)	20 (31.2)	0.060
No	249	181 (72.7)	21 (8.4)	47 (18.9)	
Experience of treating patients during any previous pandemic					
Yes	42	35 (83.3)	0 (0.0)	7 (16.7)	0.048^a^
No	271	183 (67.5)	28 (10.3)	60 (22.1)	
Attended COVID-19 related training					
Yes	148	116 (78.4)	12 (8.1)	20 (13.5)	0.003
No	165	102 (61.8)	16 (9.7)	47 (28.5)	
*Workplace related characteristics*					
Situation of working hospital					
Dhaka	165	117 (70.9)	10 (6.1)	38 (23.0)	0.153
Outside Dhaka	148	101 (68.2)	18 (12.2)	29 (19.6)	
Type of hospital					
Government	158	103 (65.2)	15 (9.5)	40 (25.3)	0.192
Private	155	115 (74.2)	13 (8.4)	27 (17.4)	
Level of hospital					
Primary	110	71 (64.5)	16 (14.5)	23 (20.9)	0.116^a^
Secondary	35	26 (74.3)	3 (8.6)	6 (17.1)	
Tertiary	168	121 (72.0)	9 (5.4)	38 (22.6)	
Department of working					
Emergency	61	44 (72.1)	9 (14.8)	8 (13.1)	0.002^a^
Medicine inpatient	75	57 (76.0)	4 (5.3)	14 (18.7)	
Surgery/gynecology inpatient	55	38 (69.1)	0 (0.0)	17 (30.9)	
ICU	15	13 (86.7)	1 (6.7)	1 (6.7)	
Infection	10	8 (80.0)	0 (0.0)	2 (20.0)	
Outpatient	56	40 (71.4)	6 (10.7)	10 (17.9)	
Others	41	18 (43.9)	8 (19.5)	15 (36.6)	
Have ICU facility					
Yes	153	113 (73.9)	8 (5.2)	32 (20.9)	0.067
No	160	105 (65.6)	20 (12.5)	35 (21.9)	
Have isolation room					
Yes	194	134 (69.1)	17 (8.8)	43 (22.2)	0.914
No	119	84 (70.6)	11 (9.2)	24 (20.2)	
Have separate donning and doffing facility					
Yes	70	51 (72.9)	3 (4.3)	16 (22.9)	0.300^a^
No	243	167 (68.7)	25 (10.3)	51 (21.0)	
Provided with appropriate PPE regularly					
Yes	149	109 (73.2)	9 (6.0)	31 (20.8)	
No	164	109 (66.5)	19 (11.6)	36 (22.0)	0.199

^a^p-value from Fisher’s exact test.

**Table 2:** Knowledge, attitude, and practice of protective measures against SARS-CoV-2 among the participants (n = 313).

Characteristics	Total	Willing,	Not willing,	Uncertain, n (%)	p-value
		n (%)	n (%)		
*Knowledge about PPE*					
Knowledge score					
Adequate (score ≥80%)	117	84 (71.8)	9 (7.7)	24 (20.5)	0.771
Inadequate (score <80%)	196	134 (68.4)	19 (9.7)	43 (21.9)	
*Attitude towards PPE*					
I understand the risks of COVID 19 pandemic for the patients and healthcare workers.					
Agree	301	213 (70.8)	28 (9.3)	60 (19.9)	0.009 ^a^
Disagree	12	5 (41.7)	0 (0.0)	7 (58.3)	
I understand how to protect myself and my patients during COVID 19 pandemic.					
Agree	265	195 (73.6)	24 (9.1)	46 (17.4)	0.000^a^
Disagree	48	23 (47.9)	4 (8.3)	21 (43.8)	
Using PPE will keep healthcare workers safe from getting COVID-19.					
Agree	247	182 (73.7)	27 (10.9)	38 (15.4)	0.000^a^
Disagree	66	36 (54.5)	1 (1.5)	29 (43.9)	
Using PPE will keep patients safe from getting COVID-19.					
Agree	188	131 (69.7)	19 (10.1)	38 (20.2)	0.600
Disagree	125	87 (69.6)	9 (7.2)	29 (23.2)	
It is convenient to use the recommended PPE.					
Agree	152	105 (69.1)	16 (10.5)	31 (20.4)	0.613
Disagree	161	113 (70.2)	12 (7.5)	36 (22.4)	
Self-reported perception of risk to be affected by COVID-19					
Low	160	130 (81.2)	6 (3.8)	24 (15.0)	0.001
High	153	88 (57.5)	22 (14.4)	43 (28.1)	
*Practice of using PPE*					
Use of PPE during patient care					
Adequate	115	93 (80.9)	6 (5.2)	16 (13.9)	0.004
Inadequate	198	125 (63.1)	22 (11.1)	51 (25.8)	
Self-reported compliance to recommended PPE					
Compliant	49	43 (87.8)	4 (8.2)	2 (4.1)	0.004^a^
Noncompliant	264	175 (66.3)	24 (9.1)	65 (24.6)	

^a^p-values from Fisher’s exact test.

### Statistical analysis

All statistical analyses were carried out using SPSS (version 22.0). Frequency distribution was used to calculate the prevalence of Willingness to work during initial lockdown due to the COVID-19 pandemic, and it was used to determine the frequency with the percentage of categorical variables while mean with standard deviation (SD) was used for continuous variables. Chi-square (χ^2^) /Fisher exact test was used to determine any difference between groups. Binary logistic regression is simply a subset or a specific type of the generalized model (GLM), and when we have categorical nominal dependent variable only two possible outcomes (success/failure), this model is used to find the predictor/s. Our outcome variable was category (Yes/No), both univariable and multivariable binary logistic regression models were used to detect the predictors of the willingness of the physicians to work during the COVID-19 pandemic. Both multiple linear and logistic regression models, there is an assumptions that there is no multicollinearity problem (dependent each to other) among the independent variables. There is no exact method to detect the multicollinearity problem in multiple logistic regression analysis. The magnitude of the standard error (SE) was used in this study to detect the multicollinearity problem among the independent variables, it was judged that there was no evidence of multicollinearity if the magnitude of the SE lied between 0.001 and 0.5 [26]. Multiple logistic regression is a model including more than one independent variable, it gives an odds ratio (OR) which is controlled for multiple confounders, the OR is known as the adjusted odds ratio (aOR) because aOR value has been adjusted for the other covariates (including confounders) [27]. The statistical significance level was set at p-value <0.05 and 95%confidence interval (CI) of odds ratio.

## Results

### Characteristics of the participants

A total of 313 physicians (74% response rate) participated in the survey. Their mean age (SD) was 30.97 (7.0) years (range 23–57 years) and 55% of them were female. Among the participants, around 74% were early-career physicians with professional experience ranged between 2 to 5 years. Almost half of the participants were working in Dhaka city (52.7%), and the same portion of them was working in tertiary level hospitals (53.7%). Half of the participants were working in the government sector (50.5%). Almost 21% of the responding physicians had an experience of treating confirmed or suspected COVID-19 patients. Around half of the participants had attended training on COVID-19 provided by the Directorate General of Health Services, Bangladesh, the World Health Organization, online education platforms of different universities like Coursera or their hospital authorities. A total of 49% of participants reported that their hospital had ICU facilities, while 62% reported having isolation room for COVID-19 patients. Only 48% of participants reported that their hospital authorities provided adequate PPE regularly (Table 1).

### Knowledge, attitude, and practice of using protective measures and self-perceived risk

Around 37% of the participants had adequate knowledge about the WHO recommended PPE and there was no significant difference of knowledge according to the willingness to work during the pandemic. Almost 96% of the participants were confident that they had understood the health risks of COVID-19 pandemic for the patients and healthcare workers, while 85% of them were confident that they had understood the need of protective measures for themselves and their patients. Almost 79% of the participants believed that proper PPE would protect healthcare providers from COVID-19, while 61% believed that it would protect the visiting patients too. Almost half of the respondents agreed that using the recommended PPE was inconvenient. Those who agreed with the statements that they understood the risk and protective measures of COVID-19 and those who believed proper PPE would protect health professionals effectively, were more likely to be willing to work during the pandemic (Table 2). It was found that almost 37% of the participants stated that they were using the recommended PPE appropriately during their regular practice, while self-reported compliance to recommended PPE was only 16%. Almost half of the physicians perceived that they were at higher risk of being infected by SARS-CoV-2 from their workplace. The adequate practice of using PPE, higher self-reported compliance, and low perceived risk were associated with willingness to report to work during the pandemic (Table 2).

### Willingness to work during pandemic

**Fig 1:**
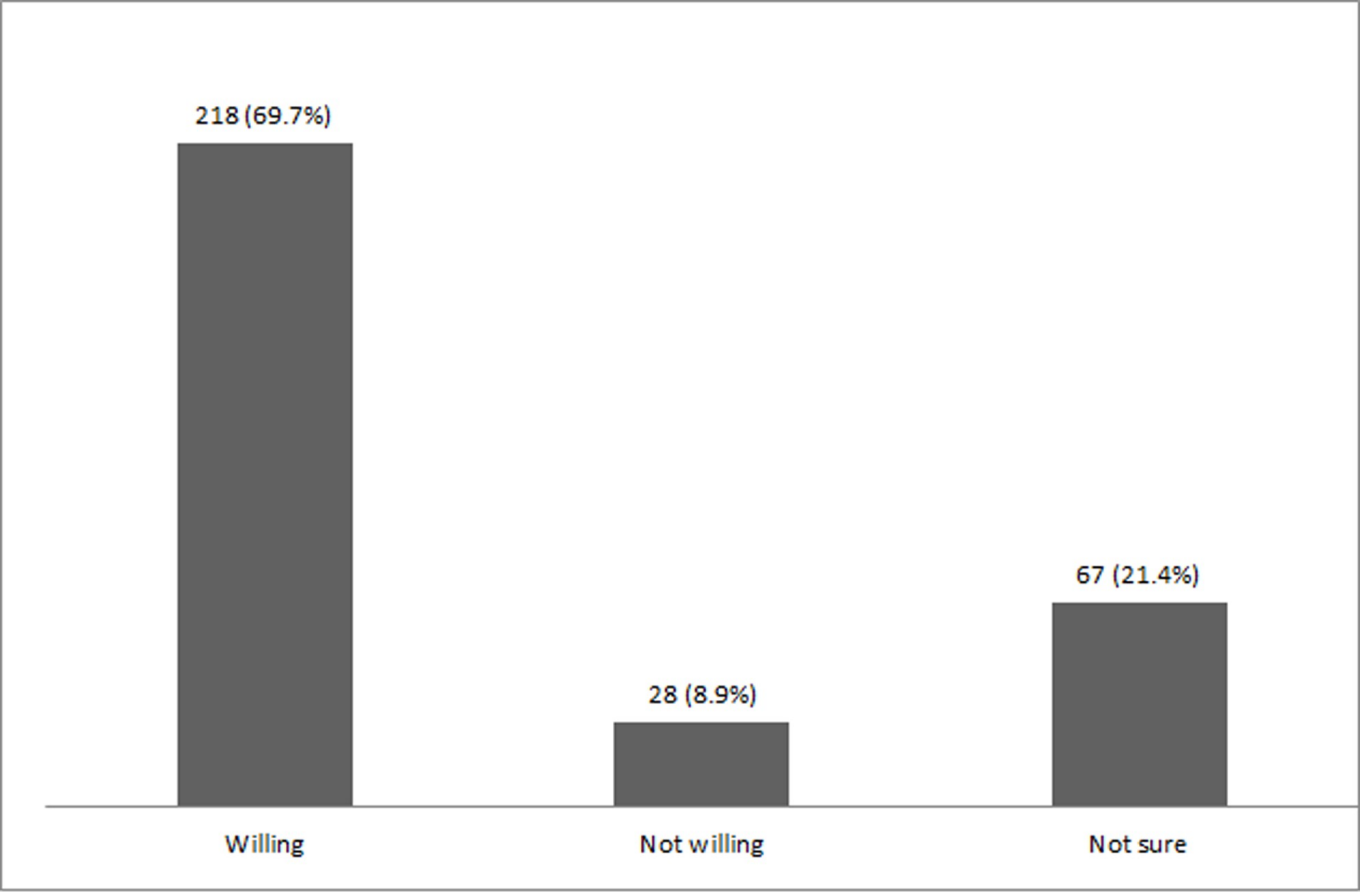
Willingness of the physicians to work during COVID-19 pandemic (n = 313).

A total of 69.7% of the participating physicians reported that they were willing to work during initial lockdown due to the COVID-19 pandemic, 8.9% reported that they were not willing, while the remaining 21.4% participants were not sure about their willingness (Fig 1).

Table 3 shows that predictors of willingness to work during pandemic (univariable and multivariable logistic regression). Though we applied both univariable and multivariable binary logistic regression models, we explained only the results came from the multivariable regression model. The magnitude value of SE for each independent variable showed that there was no evidence of multicollinearity problems among them. After controlling the effect of other factors, the multivariable logistic model demonstrated that comparatively young physicians aged 21–30 and 31–40 years had a 2.01(aOR = 2.01, 95% CI:1.20–4.32; p<0.01) and 2.11 (aOR = 2.11, 95% CI: 1.01–4.88; p<0.05) -folds higher interested respectively to work at their working place during COVID-19 pandemic than their older (age>40 years) colleagues. It was observed that physicians having experience of treating patients during previous pandemic (aOR = 8.11, 95% CI: 1.80–36.52; p<0.01), working in the emergency department (aOR = 9.92, 95% CI: 2.01–48.95; p<0.01), surgery/gynecology inpatient (aOR = 3.91, 95% CI: 1.06–14.37; p<0.05), or outpatient department (aOR = 4.53, 95% CI:1.05–19.59; p<0.05) were more interested to do willing work during COVID-19 pandemic than their counterparts. On the other hand, being a senior physician (consultant level to above) (aOR = 0.01, 95% CI: 0.01–0.10; p<0.01), and having experience of treating confirmed or suspected COVID-19 patients (aOR = 0.11, 95% CI: 0.04–0.31; p<0.01) were associated with non-willingness to work during the pandemic. Positive attitude towards the protective equipment like confidence in understanding how to protect themselves and their patients (aOR = 2.43, 95% CI: 1.01–5.85; p<0.05) and belief that using PPE would keep healthcare workers safe from getting COVID-19 (aOR = 3.13, 95% CI: 1.17–8.35; p<0.05) were also associated with the willingness to work during the pandemic. Besides these, high self-reported compliance to the recommended PPE (aOR = 6.75, 95% CI: 1.42–32.04; p<0.05) and low self-perceived risk of being infected by SARS-CoV-2 from the workplace (aOR = 2.85, 95% CI: 1.24–6.54; p<0.05) were also the predictors of willingness to report to work during COVID-19 pandemic period (Table 3). Hosmer and Lemeshow test showed that our selected model was good fitted, and the model can able to explain the variation of outcome variable by 50% (Nagelkerke R^2^- value = 0.499) (Table 3).

**Table 3:** Predictors of willingness to work during pandemic (univariable and multivariable logistic regression).

Variables		Univariate analysis			Multivariate analysis		
		cOR (95% CI)	p-value		aOR (95% CI)	p-value	
*Socio-demographic characteristics*							
Age (years)							
21–30 Vs >40^R^		2.46 (1.24–4.86)	0.010		2.01 (1.20–4.32)	0.007	
31–40 Vs >40 ^R^		2.66 (1.14–6.23)	0.024		2.11 (1.01–4.88)	0.038	
Sex							
Male Vs Female ^R^		1.87 (1.14–3.09)	0.013		1.66 (0.77–3.57)	0.188	
Marital status							
Married Vs Unmarried ^R^		0.83 (0.50–1.36)	0.461		0.98 (0.39–2.49)	0.976	
Cohabitation							
With parents Vs Alone ^R^		1.83 (0.90–3.72)	0.090		1.02 (0.38–2.75)	0.960	
With spouse only Vs Alone ^R^		1.75 (0.77–3.98)	0.178		2.26 (0.57–9.00)	0.244	
With spouse and children Vs Alone ^R^		1.06 (0.51–2.19)	0.859		0.31 (0.09–1.12)	0.075	
*Profession related characteristics*							
Professional status							
Consultant Vs Intern Physician ^R^		0.31 (0.12–0.82)	0.018		0.01 (0.01–0.10)	0.000	
Early career physician Vs Intern physician ^R^		1.08 (0.47–2.47)	0.842		0.32 (0.06–1.60)	0.168	
Professional qualification							
Graduate Vs Postgraduate ^R^		1.65 (0.98–2.79)	0.059		0.88 (0.27–2.89)	0.843	
Professional experience							
Up to 1- year Vs >5 years ^R^		1.43 (0.73–2.80)	0.288		1.06 (0.19–5.82)	0.939	
2–5 years Vs >5 years ^R^		2.60 (1.49–4.55)	0.001		4.37 (0.98–19.39)	0.052	
Experience of treating confirmed or suspected COVID-19 patients (Yes Vs No ^R^)		0.51 (0.29–0.90)	0.022		0.11 (0.04–0.31)	0.000	
Experience of treating patients during any previous pandemic (Yes Vs No ^R^)		2.40 (1.02–5.62)	0.043		8.11 (1.80–36.52)	0.006	
Attended COVID-19 related training (Yes Vs No ^R^)		2.23 (1.35–3.69)	0.002		2.00 (0.94–4.26)	0.070	
*Workplace related characteristics*							
Situation of working hospital							
Dhaka Vs Outside Dhaka ^R^		1.13 (0.70–1.83)	0.609		1.76 (0.71–4.36)	0.217	
Type of hospital							
Government Vs Private ^R^		0.65 (0.40–1.05)	0.084		0.87 (0.33–2.25)	0.776	
Level of hospital							
Primary Vs Tertiary ^R^		0.70 (0.42–1.18)	0.188		0.64 (0.18–2.21)	0.483	
Secondary Vs Tertiary ^R^		1.12 (0.49–2.57)	0.785		1.56 (0.38–6.42)	0.532	
Department of working							
Emergency Vs Others ^R^		3.30 (1.43–7.60)	0.005		9.92 (2.01–48.95)	0.005	
Medicine inpatient Vs Others ^R^		4.04 (1.79–9.12)	0.001		2.29 (0.59–8.95)	0.230	
Surgery/gynecology inpatient Vs Others ^R^		2.85 (1.23–6.62)	0.014		3.91 (1.06–14.37)	0.040	
ICU Vs Others ^R^		8.30 (1.65–41.60)	0.010		5.22 (0.63–43.10)	0.125	
Infection Vs Others ^R^		5.11 (0.96–27.00)	0.055		3.59 (0.27–46.55)	0.328	
Outpatient Vs Others ^R^		3.19 (1.37–7.44)	0.007		4.53 (1.05–19.59)	0.043	
Have ICU facility (Yes Vs No ^R^)		1.48 (0.91–2.40)	0.114		2.25 (0.72–6.98)	0.159	
Have isolation room (Yes Vs No ^R^)		0.93 (0.56–1.53)	0.777		0.81 (0.30–2.16)	0.676	
Have separate donning and doffing facility (Yes Vs No ^R^)		1.22 (0.67–2.20)	0.508		0.22 (0.08–1.59)	0.103	
Provided with appropriate PPE regularly (Yes Vs No ^R^)		1.37 (0.84–2.23)	0.199		0.73 (0.34–1.53)	0.406	
*Knowledge*, *attitude*, *and practice of protective measures against SARS-CoV-2*							
Knowledge level (Adequate Vs Inadequate ^R^)		1.17 (0.71–1.94)	0.524		1.57 (0.73–3.38)	0.247	
Attitude							
I understand the risks of COVID 19 pandemic for the patients and healthcare workers. (Agree Vs Disagree ^R^)		3.38 (1.04–10.96)	0.042		5.65 (0.72–43.92)	0.098	
I understand how to protect myself and my patients during COVID 19 pandemic. (Agree Vs Disagree ^R^)		3.02 (1.61–5.67)	0.001		2.43 (1.01–5.85)	0.047	
Using PPE will keep healthcare workers safe from getting COVID-19. (Agree Vs Disagree ^R^)		2.33 (1.33–4.09)	0.003		3.13 (1.17–8.35)	0.023	
Using PPE will keep patients safe from getting COVID-19. (Agree Vs Disagree ^R^)		1.01 (0.61–1.64)	0.988		1.24 (0.59–1.62)	0.904	
It is convenient to use recommended PPE. (Agree Vs Disagree ^R^)		0.94 (0.58–1.53)	0.831		0.77 (0.35–1.69)	0.517	
Self-perceived risk of being affected by COVID-19 (Low Vs High ^R^)		3.20 (1.92–5.33)	0.000		2.85 (1.24–6.54)	0.013	
Self-reported practice of using recommended PPE (Adequate Vs Inadequate ^R^)		2.46 (1.42–4.26)	0.001		1.37 (0.59–3.16)	0.453	
Self-reported compliance to recommended PPE (Compliant Vs Noncompliant ^R^)		3.64 (1.49–8.88)	0.004		6.75 (1.42–32.04)	0.016	
**Nagelkerke R**^**2**^- value = 0.499							
**Hosmer and Lemeshow Test**	Chi-sauare value = 14.30			p-value = 0.074			

Note: ^R^ = Reference case.

### Perceived barriers of willingness to work during pandemic

**Fig 2:**
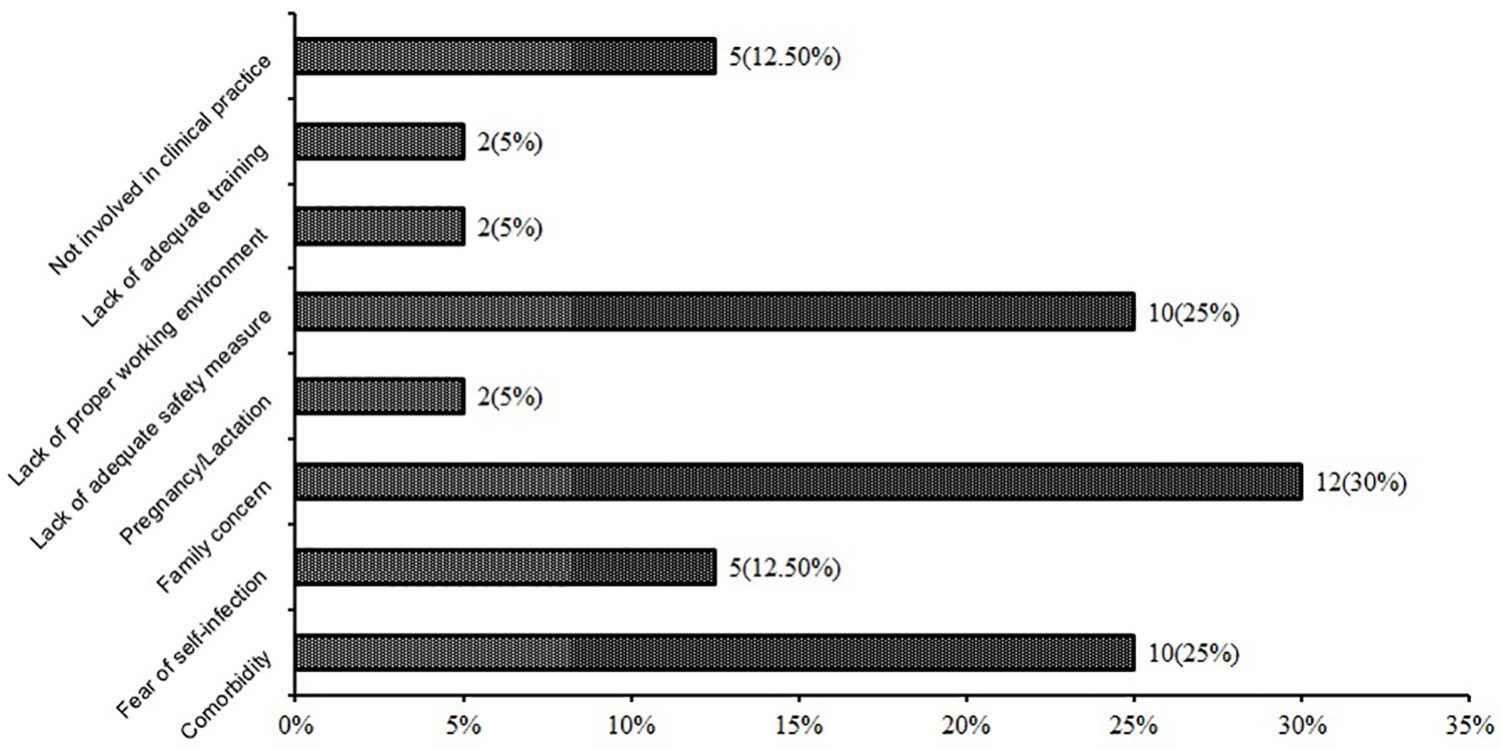
Barriers of willingness to work during pandemic (n = 40).

Out of 95 participants who were not willing to work during the pandemic or were not sure about their decision, 40 of them had reported specific causes of that, while others did not mention any specific cause. Concern for family and risk of transmitting the infection to family members from themselves were most commonly cited as a major barrier (30%) followed by having comorbidity (like bronchial asthma, diabetes, SLE, etc.) (25%) and lack of adequate safety measures (25%). Also, fear of being infected (12.2%), not involved in clinical practice (12.5%), lack of adequate training (5%), and lack of proper working environment (5%) were reported (Fig 2).

## Discussion

During the catastrophic situation of a deadly influenza pandemic, the role of the HCWs is the most crucial. Since there is an increased demand on the healthcare workforce at the time of a pandemic, their absenteeism, whether due to inability or unwillingness, becomes a major concern for the national health system. Hence, it is important to understand the personal and institutional factors that may contribute to HCWs’ informed decision to work during a pandemic considering their personal needs to keep them effectively engaged in the healthcare system. The present study attempted to explore these issues in the context of the COVID-19 pandemic among the registered physicians of Bangladesh.

Our study found that more than two-thirds of the participating physicians (almost 70%) were willing to work during the COVID-19 pandemic despite very limited resources available. Only 9% of the participants directly declined to work during the pandemic, while the decision of the remaining 21% of participants was uncertain. It was the very first study of its genre in Bangladesh according to our best knowledge. The result was comparable to the previous findings conducted in different countries, where the rate of willingness to work during a pandemic ranged between 23% and 96% depending on context [21]. For instance, during the H1N1 influenza pandemic in China, more than 82% of the HCWs expressed willingness to care for H1N1 patients [28]. The rate was 82% in Australia [29] and 85% in Japan [30], though it was comparatively low in some countries like Hong Kong (23%) [31] and Nigeria (34%) [32] during the same pandemic. Studies conducted in the context of a previous avian influenza (H5N1) pandemic reported that the rate of the willingness of the HCWs to continue work in hospitals was 84% in the USA [33], 90% in Japan [34] and 57% in Taiwan [18]. In the context of a hypothetical scenario of the influenza pandemic, the willingness of HCWs to work during pandemic ranged from 43% to 96% in different countries [21]. We also need to consider the socio-economical context of Bangladeshi physicians, availability of incentives and lack of appreciation from the people in general amid such a critical situation with inadequate decisions from the health systems.

A number of personal, institutional, and behavioral factors were found to be associated with the willingness of physicians to work during the COVID-19 pandemic. Younger physicians participating in our study were more likely to report to their willingness to work during the pandemic, which is similar to the finding of a previous study conducted in the USA [35], though some other studies found that age was not a significant factor of willingness to work [20, 36, 37]. Having multiple comorbidities makes senior physicians more vulnerable to the infection [38], which might be potentially responsible for their unwillingness to continue the job. No significant difference in willingness to work was found among male and female participants in our study. However, female HCWs were found to be more reluctant to continue their job during the pandemic in a previous review and meta-analysis [21]. Traditionally, female HCWs are socially responsible for taking care of their children and other family members, which may hinder them from continuing their job in such a complex condition.

Physicians of the departments which are perceived of not to be directly involved in handling COVID-19 patients like surgery or gynecology were more likely to report their work, though, in reality, HCWs of all the departments became similarly exposed to the infection considering the extent of the virus. Those physicians who had an experience of working during previous influenza pandemics (like H5N1 or H1N1 pandemic) were more willing to continue their work during the current pandemic, though physicians who had an experience of treating confirmed or suspected COVID-19 patients were more reluctant to continue their work. Previous exposure to a pandemic makes the HCWs more confident about their knowledge and skills, which may contribute to their willingness to work [21]. However, neither having training and level of knowledge about protective measures against the COVID-19 nor the confidence of understanding the risk of the pandemic was associated with the willingness to work, as found in our study. Despite this, some behavioral factors like self-confidence of the ability to protect themselves from the infection and positive attitude towards the efficacy of the protective measures were associated with their willingness to continue the work. Similar findings were reported by a recent meta-analysis of 41 studies conducted in different countries, where confidence on self-protection skill and perceived level of protection were associated with willingness to work during pandemics, though training and level of knowledge were also reported as significant predictors in that meta-analysis, which is contradictory with our study [21]. Though the practice of using adequate PPE or the availability of that equipment in their workplace was not associated with their willingness to work, high self-reported compliance to the recommended PPE was a predictor of that. Risk perception is an important factor to influence the HCWs’ decision to report to their job, as found both in our study and the previous ones [20, 21, 35–37]. Higher perceived risk makes them reluctant to continue their job.

Several personal and family issues such as having comorbidity that increases the vulnerability to be infected, having vulnerable members in the family like children and elderly, as well as fear of self- or family members infection by SARS-CoV-2 were the major barriers of continuing the job as reported by the physicians participating in our study during COVID-19 pandemic. Besides these institutional issues like lack of proper protective equipment, adequate training, and proper working environment were also reported as barriers of reporting to work by the physicians. These issues were also raised by the HCWs of different countries as evidenced by a number of studies during previous pandemics [21, 31, 35, 36].

Universal access to quality healthcare mostly depends on adequate human resources in the health sector. Despite the fact, there is already a large gap between the demand and availability of the HCWs in the resource-poor health care systems of low and middle-income countries like Bangladesh. A sudden rush of patients during a pandemic like COVID-19 overburdens the already stretched health care system, resulting in the ultimate collapse of the whole system. This is already being experienced by many of the affected countries, most of which are developed and high-income countries [4–6]. The effect would be more devastating in low and middle-income countries. To tackle such a catastrophe, all health care systems should have a contingency plan based on their contexts and shreds of evidence. The ability and willingness of the HCWs to continue their job during a pandemic are major components to be considered during such planning as they are the main driving force of the health system. The present study provides an insight into the physicians’ willingness and its associated factors to work during a pandemic as well as identifying the major barriers in this case for the very first time in Bangladesh, which should be considered during the policymaking.

### Strength and limitation of the study

Perhaps this was the first time we attempted to study on willingness to work during initial lockdown due to the COVID-19 pandemic among physicians in Bangladesh. However, there were some limitations of this study. Firstly, the study only included the physicians, though other HCWs including nurses, medical technicians, ambulance drivers, hospital cleaners, etc. are important stakeholders of the health care system and deserve separate investigations. Moreover, the sample size was small, and the study was conducted through online survey disseminated in social media, which may not be inclusive for those who are not using these media or unavailable during the study period. Finally, the physical ability, financial status, contextual factors relevant to the willingness of the physicians to work during pandemic were not explored, which could have yielded different structural and perceived barriers. Also, qualitative explorations of their willingness and barriers was not possible to report due to limitations attached to such online survey method. Further qualitative studies including a diverse group of HCWs is suggested for a better understanding of this issue.

## Conclusions

Our study found that the majority of the physicians of Bangladesh were willing to work during the initial lockdown period due to the COVID-19 pandemic. However, some perceived barriers were reported like transmission risk of the virus among family members, lack of personal protective equipment as well as a negative attitude towards the effectiveness of the supplied protective equipment and high perceived risk. We suggest adequate supply of PPE (personal protective equipment), institutional support to maintain recommended quarantine and isolation policy after risky hospital duty for the safety of their family members along with adequate and effective training of the physicians to provide them necessary skills to protect themselves and increase their willingness to continue the sacred duty during this crucial pandemic.

## Supporting information

S1 Data (SAV)

## List of abbreviations

HCW: Health care worker
PPE: Personal protective equipment
WHO: World Health Organization
cOR: Crude odds ratio
aOR: Adjusted odds ratio, CI: Confidence interval
